# Development and implementation of an algorithm for detection of protein complexes in large interaction networks

**DOI:** 10.1186/1471-2105-7-207

**Published:** 2006-04-14

**Authors:** Md Altaf-Ul-Amin, Yoko Shinbo, Kenji Mihara, Ken Kurokawa, Shigehiko Kanaya

**Affiliations:** 1 Department of Bioinformatics and Genomics, Graduate School of Information Science, Nara Institute of Science and Technology (NAIST), 8916-5 Takayama, Ikoma, Nara 630–0101, Japan

## Abstract

**Background:**

After complete sequencing of a number of genomes the focus has now turned to proteomics. Advanced proteomics technologies such as two-hybrid assay, mass spectrometry etc. are producing huge data sets of protein-protein interactions which can be portrayed as networks, and one of the burning issues is to find protein complexes in such networks. The enormous size of protein-protein interaction (PPI) networks warrants development of efficient computational methods for extraction of significant complexes.

**Results:**

This paper presents an algorithm for detection of protein complexes in large interaction networks. In a PPI network, a node represents a protein and an edge represents an interaction. The input to the algorithm is the associated matrix of an interaction network and the outputs are protein complexes. The complexes are determined by way of finding clusters, i. e. the densely connected regions in the network. We also show and analyze some protein complexes generated by the proposed algorithm from typical PPI networks of *Escherichia coli *and *Saccharomyces cerevisiae*. A comparison between a PPI and a random network is also performed in the context of the proposed algorithm.

**Conclusion:**

The proposed algorithm makes it possible to detect clusters of proteins in PPI networks which mostly represent molecular biological functional units. Therefore, protein complexes determined solely based on interaction data can help us to predict the functions of proteins, and they are also useful to understand and explain certain biological processes.

## Background

Large-scale experiments are producing huge data sets of protein-protein interactions making it increasingly difficult to visualize and analyze the information contained in these data [1]. Being able to apply computational methods can alleviate a lot of problems in this regard. Therefore, a general trend is to represent the interactions as a network/graph and to apply suitable graph algorithms to extract necessary information. In the post-genomic era, one of the most important issues is to find protein complexes from the protein-protein interaction (PPI) networks. Protein complexes can help us to predict the functions of proteins [2], and they are also useful to understand and explain certain biological processes. The results obtained from different technologies for detection of high-throughput protein-protein interactions such as yeast two hybrid assay (Y2H) and mass spectrometry of purified complexes, say tandem affinity purification (TAP) [3] and high-throughput mass-spectrometric protein complex identification (HMS-PCI) [4] show some variations. For example, the common PPI between the two different mass-spectrometry approaches stands at 1,728 pairs, which correspond to 27.5% of PPI detected by TAP and 19.2% of PPI detected by HMS-PCI. These variations imply that many of the experimentally determined interactions might be false positives or the experiments are not complete yet. Hence, generation of protein complexes based on interaction networks of separate or combined data sets is helpful because the interactions that are involved in complexes are likely to be true.

In the present study, we assume that the interaction network is an undirected simple graph. A graph is undirected if its edges are not directed and a graph is simple if it has no parallel edge or self loop. It is suggested that clusters or locally dense regions of an interaction network represent protein complexes. However, the term "locally dense region" implies a very flexible concept. Some well-known clustering methods are *k*-core, *k*-block, *k*-plex and *n*-clan clustering [5-7]. These strategies are based on the number of node degrees or the number/length of paths between two nodes within the cluster. A *k*-core is a maximal subgraph such that each node in the subgraph has at least degree *k*. A *k*-plex is a subgraph such that each node in the subgraph has at least degree |*N*| - *k*, where |*N*| is the size of the subgraph. A *k*-block is a maximal subgraph such that each pair of nodes in the subgraph is connected by *k *node-disjoint paths. An *n*-clan is a subgraph such that the distance between any two nodes is not greater than *n *for paths within the subgraph. Generating clusters based on fixed values of *n *or *k *is too restrictive and is not very helpful for detecting protein complexes in interaction networks.

Already a number of approaches have been proposed for detection of protein complexes in PPI networks. The sequential constructive method of [1] makes use of the concepts of clustering coefficient and k-core graphs. Another approach described in [8] use hierarchical clustering. However they introduced the concept of secondary distances instead of considering the path length as the distance between a pair of proteins because of the fact that such distances among proteins are constrained and often cause distance ties. The approach of [9] starts by composing an initial random clustering and then iteratively moving one node from one cluster to another in a randomized fashion to improve the clustering's cost. Once the clusters are generated, they are filtered based on cluster size, density and functional homogeneity keeping in mind the criteria of the known biological complexes. Another approach related to analyzing protein complexes is super-paramagnetic clustering [10].

**Figure 1:**
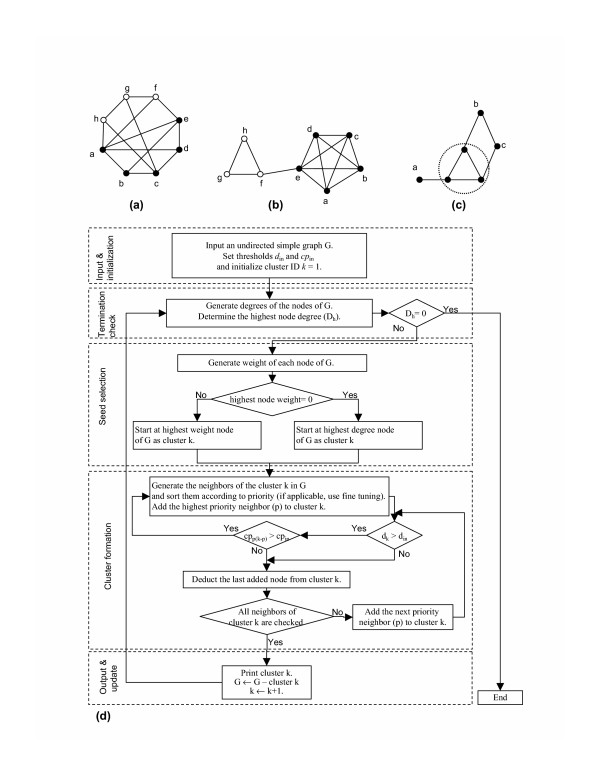
Concepts of the proposed method. (a) and (b) Two typical graphs of the same size and density. (c) A typical cluster and its neighbors. (d) Flow-chart of the proposed algorithm.

By intuition we realize that densely connected regions of a graph are clusters. However ensuring density alone is not enough. The graphs of Fig. 1(a) and 1(b) consists of 8 nodes each and both are of density 0.5 (a formal definition of density is in the next section). But Fig. 1(a) seems to be a single cluster while Fig. 1(b) is divided into two clusters consisting of node sets {*a, b, c, d, e*} and {*f, g, h*}. The proposed algorithm can tackle this problem by keeping track of the periphery of a cluster by monitoring cluster property (a formal definition of cluster property is in the next section) of a neighbor with respect to a cluster. Therefore, in short the algorithm detects densely connected regions of a graph that are separated by sparse regions. As a whole we consider that clusters are local phenomena in a network (if we imagine the presence of non-overlapping clusters) and we find that periphery is a property of a cluster. Also density is an overall measure of cohesiveness of the nodes of a cluster. Therefore we used the concepts of density and periphery tracking in the proposed algorithm. It is likely that two nodes that belong to the same cluster have more common neighbors than two nodes that do not. We used this notion in seed selection and priority node selection in the cluster formation process.

### The proposed algorithm

#### Terminology

A protein-protein interaction network is considered as an undirected simple graph G = (*N*, *E*) that consists of a finite set of nodes *N *and a finite set of edges *E*. Before details of the algorithm, we define some terminologies used in this paper.

#### Definition 1

***Density ****d*_*k *_of any cluster *k *is the ratio of the number of edges present in the cluster (|*E*_*k*_|) and the maximum possible number of edges in the cluster (|*E*_*k*_|_*max*_) and is represented by (1).

dk=|Ek||Ek|max⁡=2×|Ek||Nk|×(|Nk|−1)     (1)
 MathType@MTEF@5@5@+=feaafiart1ev1aaatCvAUfKttLearuWrP9MDH5MBPbIqV92AaeXatLxBI9gBaebbnrfifHhDYfgasaacH8akY=wiFfYdH8Gipec8Eeeu0xXdbba9frFj0=OqFfea0dXdd9vqai=hGuQ8kuc9pgc9s8qqaq=dirpe0xb9q8qiLsFr0=vr0=vr0dc8meaabaqaciaacaGaaeqabaqabeGadaaakeaacqWGKbazdaWgaaWcbaGaem4AaSgabeaakiabg2da9maalaaabaWaaqWaaeaacqWGfbqrdaWgaaWcbaGaem4AaSgabeaaaOGaay5bSlaawIa7aaqaamaaemaabaGaemyrau0aaSbaaSqaaiabdUgaRbqabaaakiaawEa7caGLiWoadaWgaaWcbaGagiyBa0MaeiyyaeMaeiiEaGhabeaaaaGccqGH9aqpdaWcaaqaaiabikdaYiabgEna0oaaemaabaGaemyrau0aaSbaaSqaaiabdUgaRbqabaaakiaawEa7caGLiWoaaeaadaabdaqaaiabd6eaonaaBaaaleaacqWGRbWAaeqaaaGccaGLhWUaayjcSdGaey41aqRaeiikaGYaaqWaaeaacqWGobGtdaWgaaWcbaGaem4AaSgabeaaaOGaay5bSlaawIa7aiabgkHiTiabigdaXiabcMcaPaaacaWLjaGaaCzcamaabmaabaGaeGymaedacaGLOaGaayzkaaaaaa@5F9C@

Here, |*N*_*k*_| is the size of the cluster, i. e. the number of nodes in the cluster. The density of a cluster is a real number ranging from 0 to 1.

#### Definition 2

The ***cluster property ****cp*_*nk *_of any node *n *with respect to any cluster *k *of density *d*_*k *_and size |*N*_*k*_| is defined by (2).

cpnk=|Enk|dk×|Nk|     (2)
 MathType@MTEF@5@5@+=feaafiart1ev1aaatCvAUfKttLearuWrP9MDH5MBPbIqV92AaeXatLxBI9gBaebbnrfifHhDYfgasaacH8akY=wiFfYdH8Gipec8Eeeu0xXdbba9frFj0=OqFfea0dXdd9vqai=hGuQ8kuc9pgc9s8qqaq=dirpe0xb9q8qiLsFr0=vr0=vr0dc8meaabaqaciaacaGaaeqabaqabeGadaaakeaacqWGJbWycqWGWbaCdaWgaaWcbaGaemOBa4Maem4AaSgabeaakiabg2da9maalaaabaWaaqWaaeaacqWGfbqrdaWgaaWcbaGaemOBa4Maem4AaSgabeaaaOGaay5bSlaawIa7aaqaaiabdsgaKnaaBaaaleaacqWGRbWAaeqaaOGaey41aq7aaqWaaeaacqWGobGtdaWgaaWcbaGaem4AaSgabeaaaOGaay5bSlaawIa7aaaacaWLjaGaaCzcamaabmaabaGaeGOmaidacaGLOaGaayzkaaaaaa@493B@

Here, |*E*_*nk*_| is the total number of edges between the node *n *and each of the nodes of cluster *k*.

In Fig. 1(a), the cluster property of node *f *with respect to cluster {*a, b, c, d, e*} is 20.7×5
 MathType@MTEF@5@5@+=feaafiart1ev1aaatCvAUfKttLearuWrP9MDH5MBPbIqV92AaeXatLxBI9gBaebbnrfifHhDYfgasaacH8akY=wiFfYdH8Gipec8Eeeu0xXdbba9frFj0=OqFfea0dXdd9vqai=hGuQ8kuc9pgc9s8qqaq=dirpe0xb9q8qiLsFr0=vr0=vr0dc8meaabaqaciaacaGaaeqabaqabeGadaaakeaadaWcaaqaaiabikdaYaqaaiabicdaWiabc6caUiabiEda3iabgEna0kabiwda1aaaaaa@338B@ ≈ 0.57 while in Fig. 1(b) the cluster property of node *f *with respect to cluster {*a, b, c, d, e*} is 11×5
 MathType@MTEF@5@5@+=feaafiart1ev1aaatCvAUfKttLearuWrP9MDH5MBPbIqV92AaeXatLxBI9gBaebbnrfifHhDYfgasaacH8akY=wiFfYdH8Gipec8Eeeu0xXdbba9frFj0=OqFfea0dXdd9vqai=hGuQ8kuc9pgc9s8qqaq=dirpe0xb9q8qiLsFr0=vr0=vr0dc8meaabaqaciaacaGaaeqabaqabeGadaaakeaadaWcaaqaaiabigdaXaqaaiabigdaXiabgEna0kabiwda1aaaaaa@31AB@ = 0.2. A higher value of cluster property of a neighbor indicates that it is part of the cluster while a lower value indicates that it is part of the periphery. The graph of Fig. 1(b) can be separated into two clusters by using the concept of cluster property.

#### Definition 3

The ***weight w***_*uv*_***of an edge ***(*u, v*) ∈ *E *is the number of the common neighbors of the nodes *u *and *v*.

#### Definition 4

The ***weight w***_*n *_***of a node ****n *is the sum of the weights of the edges connected to the node i.e. *w*_*n *_= ∑ *w*_*nu *_for all *u *such that (*n, u*) ∈ *E*.

### The flow-chart of the algorithm

The flowchart of the algorithm is shown in Fig. 1(d) and it is divided into five major steps: Input & initialization, Termination check, Seed selection, Cluster formation and Output & update. We now discuss each step.

#### Input & initialization

The input to the algorithm is an undirected simple graph and hence the associated matrix of the graph is read first. It is also necessary to provide a value of minimum density we allow for the generated clusters and a minimum value for cluster property that determines the nature of periphery tracking. From now on, these input values of density and cluster property will be referred to as *d*_*in *_and *cp*_*in *_respectively. Clustering can be performed several times using different input values for *d*_*in *_and *cp*_*in*_, which allows the suitable set of clusters to be chosen from among a number of options. The cluster ID, *k *is initialized to 1.

#### Termination check

Once a cluster is generated, it is removed from the graph. The next cluster is then formed in the remaining graph and the process goes on until no edge is left in the remaining graph. For a graph with no edge, the degree of each node is zero. When such situation arrives, the algorithm terminates.

#### Seed selection

Each cluster starts at a deterministic single node which we call the seed node. The highest weight node is considered as the seed node. However, if the highest node-weight is zero, the highest degree node is considered as the seed node. The weights of nodes are determined by summing up the weights of incident edges and the weights of edges are calculated by matrix multiplication. Let *M *be the associated matrix of *G*. Obviously the dimension of *M *is |*N*|. It can be proved that (*M*^2^)_*uv *_for *u ≠ v *represents the number of paths of length two between the nodes *u *and *v*. A little thought reflects that this is actually the number of common neighbors of the nodes *u *and *v*, i.e. the weight of the edge (if one exists) between *u *and *v*. The timing complexity of matrix multiplication is of the order *O*(|*N*|^3^). However, we need only those elements of *M*^2 ^for which *M*_*uv *_= 1. So the complexity of finding weights of the edges can be reduced to *O*(|*N*| × |*E*|). The weight of every node is calculated by summing up the weights of its connecting edges and the complexity of finding the weights of all the nodes is of the order O(|*N*|^2^). The highest weight node is then determined as the seed.

#### Cluster formation

The cluster starts as a single node and then grows gradually by adding nodes one by one from its neighbors. The neighbors of a cluster are the nodes connected to any node of the cluster but not part of the cluster. It is very important to add priority neighbors to the cluster first to guide the cluster formation in a proper way. The priority is determined based on two measures: (1) the sum of the weights of the edges between a neighbor and each of the nodes of the cluster and (2) the number of edges between a neighbor and each of the nodes of the cluster. Therefore, a double sorting is performed to sort the neighbors. The running time to perform this job is of the order O(max(|*N*| × |*N*_*k*_|, |*N*_*n*_|^2^)), where |*N*_*k*_| is the size of the cluster and |*N*_*n*_| is the size of the neighbors. Therefore, the worst case running time for this job would be polynomial of the order |*N*|^2^.

Furthermore, we use some fine-tuning in the sorting process when cluster length is more than one but all neighbors are connected to the cluster by only single edge. In the following example we explain the purpose of fine-tuning. Fig. 1(c) shows a dotted-line encircled cluster, say at some instant of the cluster formation process, and its neighbors *a, b *and *c*. All three neighbors are of equal priority if sorting is performed according to the two measures mentioned above. However, by common sense we realize that *b *or *c *should be given more priority. The fact is that other than the single edge link with the cluster, *b *or *c *is also connected to the cluster by a link of length 2 that goes outside of the cluster. Based on this fact, we fine-tune the sorting of the neighbors such that *b *or *c *comes up as the highest priority neighbor. However when fine-tuning is used to sort the neighbors, we use half the value of *cp*_*in *_for periphery checking and thus help to form some sparse clusters. The timing complexity of the fine-tuning process is of the order O(|*N*| × |*N*_*k*_| × |*N*_*n*_|). Theoretically, the value of |*N*_*k*_| or |*N*_*n*_| can approach near to |*N*| but |*N*_*k*_| + |*N*_*n*_| ≤ |*N*|. In most practical cases |*N*_*k*_| and |*N*_*n*_| can be considered as constants. Furthermore, the fine-tuning process is performed only under special conditions and not every time when a node is added to a cluster.

In a simple graph the number of paths of length 1 between any two nodes is at best 1. The number of common neighbors between any two nodes is actually equal to the number of paths of length 2 between them. In fact, we consider the paths of length 1 and length 2 to determine the priority of a neighbor with respect to a cluster. In case more than one neighbor are of equal highest priority, we choose any one of them as the highest priority node while sorting. The performance of the sorting can somewhat be improved by taking into consideration the paths of length 3 and 4 and so on. However, that increases the computational burden substantially. In the present work, we do not consider this and the proposed algorithm works well for graphs that have a nice cluster structure, i. e. the graphs that have densely connected regions separated by sparse regions.

We check two things before adding a node to a cluster. First, we make sure that addition of the node to the cluster does not cause the density *d*_*k *_of the cluster to fall below *d*_*in*_, the input density. Second, we check whether the node is part of the cluster or part of the periphery. If a node is part of the cluster it should be connected to a reasonable number of edges within the cluster. For example for a cluster of density *d_k_*, each node on an average should be connected to *d*_*k*_× (|*N*_*k*_| - 1) edges within the cluster, where |*N*_*k*_| is the size of the cluster. We do not add a neighbor to a cluster if its cluster property is less than *cp*_*in*_. We can choose the value of *cp*_*in *_from within the range 0 <*cp*_*in*_≤ 1.

#### Output & update

Once a cluster is generated, it is printed and graph *G *is updated by removing the present cluster, i.e. the nodes belonging to the present cluster and the incident edges on these nodes are removed from *G*. The cluster ID, *k *is updated by adding 1 to it.

The procedure for generating and sorting the neighbors is performed every time a node is added to a cluster. The total number of nodes in the network is |*N*| and therefore, the worst-case timing complexity of the algorithm is polynomial of the order O(|*N*|^3^).

### Generation of overlapping clusters

The algorithm represented by the flow-chart of Fig. 1(d) generates non-overlapping clusters because once a cluster is generated it is removed from the graph and the next cluster is generated in the remaining graph. However, we can also generate overlapping clusters based on the same clustering concept. To do so, we extend the generated non-overlapping clusters by adding nodes to them from among their first neighbors in the original graph (not in the remaining graph). In this process, the restrictions of threshold density and periphery property are similarly conserved.

## Results and discussion

We applied the proposed algorithm to typical PPI networks of *E. coli, S. cerevisiae *and a random network. In the following subsections the results are discussed from different angles of consideration.

### Complexes in the *E. coli *PPI network

**Figure 2:**
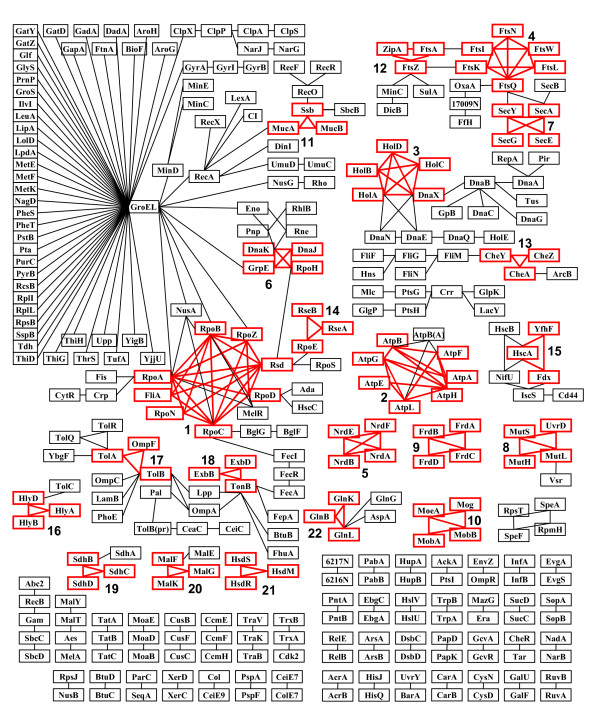
A protein-protein interaction network of *E. coli *showing high-density complexes of size ≥ 3 detected by the proposed algorithm. Functions of the proteins of 10 largest complexes are as follows: complex 1, components of RNA polymerase (RpoA, RpoB, RpoC, Rsd, RpoZ RpoD, RpoN, FliA); complex 2, components of ATP synthetase (AtpA, AtpB, AtpE, AtpF, AtpG, AtpH, AtpL); complex 3, components of DNA polymerase (DnaX, HolA, HolB, HolD, and HolC); complex 4, proteins involved in cell division (FtsQ, FtsI, FtsW, FtsN, FtsK and FtsL); complex 5, components of ribonucleoside-diphosphate reductase (NrdA, NrdB, NrdE, NrdF); complex 6, chaperons (DnaK, GrpE, DnaI) and a heat-shock sigma factor (RpoH), complex 7, component of protein translocase (SecA, SecE, SecY, SecG); complex 8, DNA mismatch repair proteins (MutL, MutS, MutH, UvrD); complex 9, components of fumarate reductase (FrdA, FrdB, FrdC, FrdD); and complex 10, proteins associated with molybdopterin biosynthesis (MoeA, MoeB, MobB, Mog). 11 to 22 are 3-protein complexes, which mostly consist of similar function proteins.

The network of *E. coli *proteins consists of 363 interactions involving a total of 336 proteins as shown in Fig. 2. We have collected these interactions from DIP [11]. This is not too big a network and we have chosen this to demonstrate the performance of the algorithm through visualization. Twenty-two complexes of size ≥ 3 are obtained when clustering is performed using *d*_*in*_*=*0.70 and *cp*_*in*_*=*0.50 with non-overlapping mode, which are shown connected by thick red edges (in other words, each complex is separated from its surroundings, by thin black edges) in Fig. 2. It is easy to check that the algorithm very effectively separates densely connected regions of the graph. It is important to note that each of the complexes contains mostly similar function proteins. For the sake of comparison, by applying k-core clustering, we find that the highest k-core of this network is a 5-core sub-graph consisting of RpoA, RpoB, RpoZ, RpoC, RpoD and Rsd. This is similar to complex 1 of Fig 2 i.e. the highest k-core can detect only one complex. The 4-core sub-graph is consisting of GroEL, RpoA, RpoB, RpoZ, NusA, RpoC, RpoD, DnaX, Rsd, HolA, HolB, HolD, HolC, RpoN and FliA, which roughly encompasses the complex 1 and complex 4 and therefore some other algorithm is necessary to isolate them. The other k-core (k<4) sub-graphs further complicate the situation. So from Fig. 2 it is evident that the proposed graph clustering is useful for discretely extracting molecular biological functional units in protein-protein interaction networks.

### Comparison of PPI and random networks

**Figure 3:**
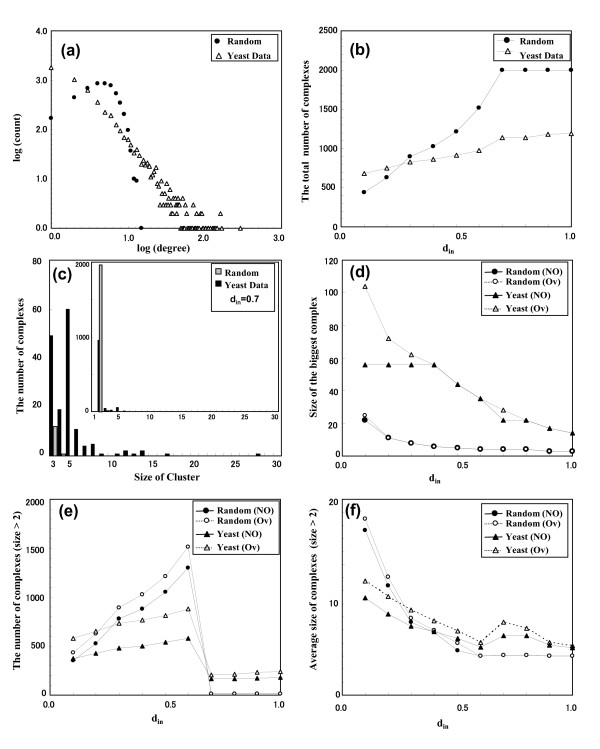
Comparison of yeast PPI network with a random network in the context of (a) degree distribution, (b) total number of complexes, (c) distribution of the complexes generated using d_*in *_= 0.7 with respect to size, (d) size of the biggest complex, (e) the number of complexes of size ≥ 3 and (f) the average size of the complexes of size ≥ 3. We generated 10 overlapping (Ov) and 10 non-overlapping (NO) sets of complexes by using 10 different values of d_*in *_for both the networks. We then calculated the aforementioned quantities for each of the generated sets and plotted them.

Producing different but statistically reasonable outputs when the algorithm is applied to different type of networks would be supportive in favor of the performance of the algorithm. Hence we compare PPI and random networks in terms of number and size of complexes in them generated by the present algorithm. For this purpose, we use the yeast *S. cerevisiae *PPI network because unlike the *E. coli *network of Fig. 2, it contains a large number of protein-protein interactions which is good enough for statistical analysis. We extract a set of 12487 unique binary interactions involving 4648 proteins by discarding self-interactions of the PPI data obtained from [12]. For the sake of rational comparison, we prepared a random graph of the same size (consisting of the same number of nodes and edges) of the yeast PPI network. Erdös and Rényi first studied random networks in the late 1950s and showed that the degree distribution of a random network for a large |*N*| approximately follows Poisson's distribution (*p*(*k*) = *e*^-λ^λ^*k*^/*k*!) [13,14]. It is reported that the degree distribution of a PPI network follows the power law (*p*(*k*) ~*k*^-*r*^) [15]. Fig. 3(a) shows the degree distribution of the *S. cerevisiae *PPI network and that of the generated random network used in this work (plotted using log-log scale). The degree distribution of the random network in Fig. 3(a) resembles Poisson's distribution, which is consistent with Erdös-Rényi network model. The degree distribution of the yeast PPI network is consistent with the power law distribution.

In total we generated 10 overlapping and 10 non-overlapping sets of complexes by using *cp*_*in*_*=*0.5 and *d*_*in *_= 0.1, 0.2, 0.3,...,0.9 or 1.0 for both the PPI and the random networks. Out of these, the complexes concerning the yeast PPI will be referred to as '20 sets of yeast complexes' in the rest of this paper. Fig 3(b) shows the relation between *d*_*in *_and the total number of complexes. The random graph has more complexes than the yeast PPI in the region *d*_*in*_*>*0.3 (Fig. 3(b)). The distribution of the complexes generated using *d*_*in *_= 0.7 is shown in Fig. 3(c). 2-protein complexes are much more in the random graph. However bigger complexes (size ≥ 3) are much more in the yeast PPI. It is further evident from Fig. 3(d) that most of the high-density complexes in the random graph are simple complexes consisting of 2 or 3 nodes each because the size of the biggest complex in the high-density region is very small. Even in the low-density region, the size of the biggest cluster is much lower compared to that of the yeast PPI network indicating that there is almost no cluster structure in the random graph. Also we calculated and found that the traditional clustering coefficient (an average measure of the level of interconnection among the neighbors of a node in the context of the whole network) of the random network (0.0012) is much lower than that of the yeast PPI network (0.1801). A cluster of size 2 that represents just one interaction is a trivial cluster. Hence, we counted the clusters of size ≥ 3 in both the random and the PPI networks (Fig. 3(e)). Many 3-protein complexes with 2 edges (density 0.66) are generated when *d*_*in*_*=*0.6 is used. Such 3-protein complexes are far more common in random graphs. This is evident from the sudden drop after *d*_*in*_*=*0.6, because when *d*_*in*_*=*0.7 is used the aforementioned type of 3-protein complexes are not generated. The average size of the generated complexes is higher in low-density regions but it is lower in high-density regions for both the random and the yeast PPI network (Fig. 3(f)). However, for random graph it is higher than that of the yeast PPI network in low-density region but very rapidly decreases with increasing density and eventually becomes lower (~3) compared to that of the yeast PPI network. This trend also implies that the cluster structure is almost absent in the random graph.

### The effect of *cp_in _*on clustering

**Figure 4:**
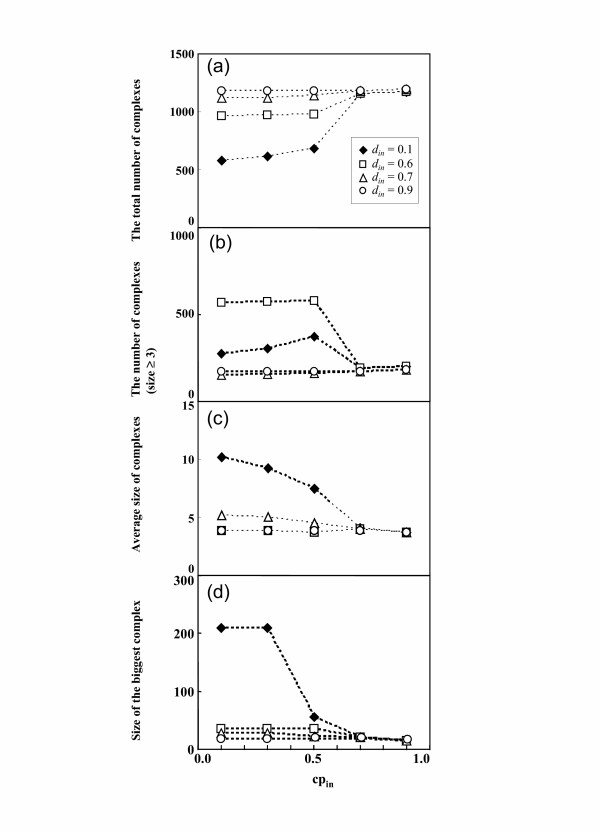
The effect of *cp*_*in *_on clustering. Twenty sets of non-overlapping clusters are generated from the yeast PPI using *cp*_*in *_= 0.1, 0.3, 0.5, 0.7, 0.9 and for each of these values of *cp*_*in *_using *d*_*in *_= 0.1, 0.6, 0.7, 0.9. We then calculated and plotted (a) the total number of clusters, (b) the number of clusters of size ≥ 3, (c) the average size of the clusters of size ≥ 3, and (d) the size of the biggest cluster with respect to *cp*_*in*_.

We used *cp*_*in*_*=*0.5 for the experiments discussed in previous sections. However the variation of *cp*_*in *_can also affect the outcome of the clustering. To observe the overall effects of *cp*_*in*_, we utilized the PPI network of yeast because it is reasonably big. From Fig. 4, it is evident that if very high *cp*_*in *_is used the variation of *d*_*in *_does not affect much. Similarly, if very high *d*_*in *_is used, the variation of *cp*_*in *_does not have much effect. If high value is used for either *cp*_*in *_or *d*_*in*_, the generated clusters are of high density but smaller in size and hence relatively more in number (Fig. 4(a)). However, many such clusters are trivial clusters consisting of only two proteins and therefore the number of clusters of size ≥ 3 are lower in sets generated using high values for *cp*_*in *_or *d*_*in *_(Fig. 4(b)). The highest number of clusters of size ≥ 3 is obtained in case of *cp*_*in*_*=*0.5 and *d*_*in*_*=*0.6. Many clusters in this case are 3-protein clusters of density 0.66. The size of the biggest cluster and the average size of the clusters are larger when both *cp*_*in *_and *d*_*in *_are low and these values are low when either *cp*_*in *_or *d*_*in *_is high (Fig 4(c)) and 4(d)). For *cp*_*in*_*<*0.5 the variation of *d*_*in *_has noticeable effect on clustering and this effect quickly reduces for *cp*_*in*_*>*0.5 in the case of the yeast PPI. In general, from the periphery tracking point of view, we consider that a reasonable and balanced value for *cp*_*in *_is 0.5 because it is in the middle of the parameter space. However it can be said that the larger the value of *cp*_*in *_the more spherical the structure of the generated complexes.

### Analysis on the predicted complexes of yeast

Some members of the 20 sets of yeast complexes (mentioned in the section entitled "Comparison of PPI and Random Networks") are common in more than one set and some are unique to a single set. Each of the generated complexes may have its own significance. However it is beyond the scope of this paper to discuss the biological significance of all these complexes (available at [16]). Here, we compare the predicted complexes with known complexes to find out how they match, evaluate the quality of the predicted complexes in a general sense and describe a bit detail of a group of specific complexes.

### Comparison with the known complexes

**Figure 5:**
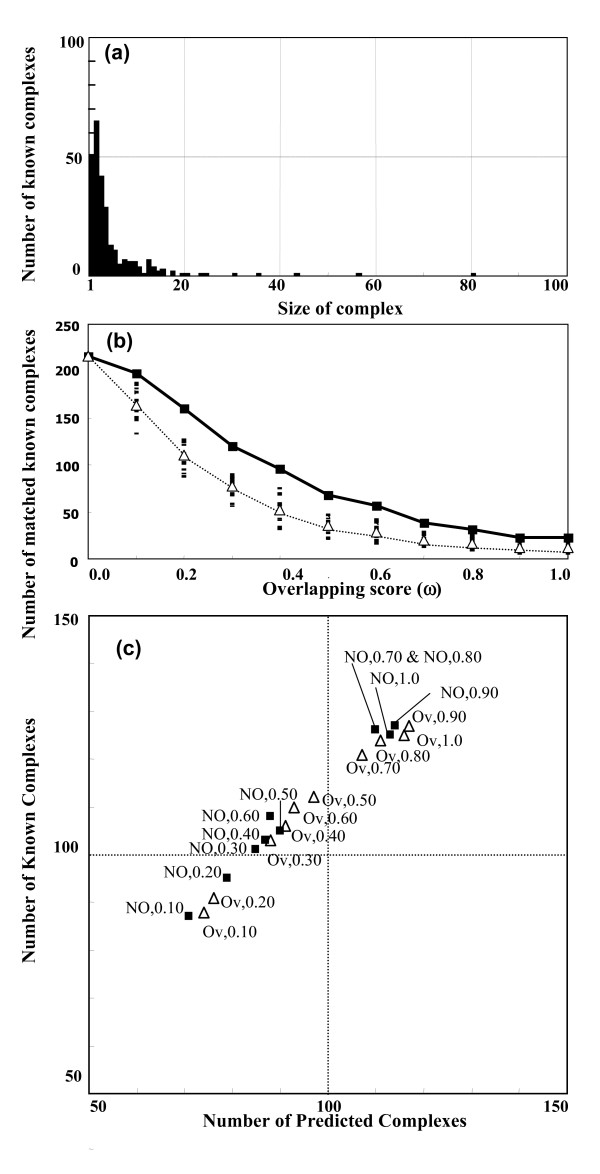
Comparison of predicted complexes with known complexes. (a) Distribution of known complexes with respect to size. (b) Number of matched known complexes with respect to the minimum overlapping score for all 20 sets. The triangular dots (connected by dotted line) show the average and the solid square dots (connected by thick solid line) show the union of the matched known complexes. (c) Number of matched known complexes with respect to number of predicted complexes for 20 sets. Each point is labeled by corresponding mode of clustering, i. e. non-overlapping (NO) or overlapping (Ov) and *d*_*in *_value.

We obtained a list of known complexes together with constituent protein names from [17]. The distribution of these complexes with respect to size is shown in Fig. 5(a), which implies that many of the known complexes are small in size. There are a total of 317 manually annotated complexes but for the present experiments we consider only 216 complexes that consists of two or more proteins each. We use the same scoring scheme used in [1] to determine how effectively a predicted complex matches a known complex. The overlap score between a predicted and a known complex is calculated by using (3).

ω=i2a×b     (3)
 MathType@MTEF@5@5@+=feaafiart1ev1aaatCvAUfKttLearuWrP9MDH5MBPbIqV92AaeXatLxBI9gBaebbnrfifHhDYfgasaacH8akY=wiFfYdH8Gipec8Eeeu0xXdbba9frFj0=OqFfea0dXdd9vqai=hGuQ8kuc9pgc9s8qqaq=dirpe0xb9q8qiLsFr0=vr0=vr0dc8meaabaqaciaacaGaaeqabaqabeGadaaakeaacqaHjpWDcqGH9aqpdaWcaaqaaiabdMgaPnaaCaaaleqabaGaeGOmaidaaaGcbaGaemyyaeMaey41aqRaemOyaigaaiaaxMaacaWLjaWaaeWaaeaacqaIZaWmaiaawIcacaGLPaaaaaa@3A83@

Here, *i *is the size of the intersection set of a predicted complex with a known complex, *a *is the size of the predicted complex and *b *is the size of the known complex. In Fig. 5(b), we show the plot of the number of matched known complexes with respect to the minimum overlap score for the 20 sets of yeast complexes. In [1] it is assumed that a predicted complex more or less matches a known complex if its overlapping score is above 0.2. In Fig. 5(b), the best result at overlapping score ω = 0.2 (127 matches) is obtained for two sets generated using *d*_*in*_*=*0.9 with overlapping and non-overlapping modes. Complexes produced by using high *d*_*in *_are many in number but smaller in size and most of the known complexes are also of small size (Fig. 5(a)). Hence best matching is obtained for sets generated using high *d*_*in *_values. However, the union of the matched known complexes for all the sets denoted by solid square dots are reasonably larger than matched known complexes of any single set and not all but many of the 20 sets have unique contributions to it. Therefore generating complexes using different values of *d*_*in *_is useful for protein complex prediction. However, concerning a single set more matching can be expected for a set produced using high *d*_*in *_value.

For each of the sets, the number of matched known complexes is somewhat higher than the number of corresponding predicted complexes. This implies that in some cases more than one known complexes match with a single predicted complex. Fig. 5(c) illustrates this for complexes having an overlapping score above 0.2. A similar trend has been reported in [1].

The outcome of the prediction greatly depends on the input network. In general it can be suggested that the larger the amount of interaction data the larger the amount of information contained in the network and the better the predictions. We applied our algorithm to MIPS (Munich Information center for Protein Sequences) interaction data (12487 interactions involving 4648 proteins). In [1] one of the data sets consists of 15143 interactions involving 4825 proteins collected from MIPS and several other sources. Both of these data sets are not exactly the same and a direct comparison of the results is not possible. However, with any single combination of parameters, in [1] 63 MIPS complexes have been predicted with overlapping score above 0.2 while in the present work 127 MIPS complexes have been predicted with overlapping score above 0.2. So far we realize, 840 parameter combinations were explored to find out the best combination in [1], while we explored only 20 combinations. Therefore, ensuring density and simultaneously checking periphery is a suitable strategy to find out protein complexes from interaction networks.

### Overall quality of the predicted complexes

It has been stated in [2] that 65% of 2709 interactions (involving a total of 2039 proteins) occurred between protein pairs with at least one common function. Similar results have been reported in [18]. Though the majority of the interactions are between similar function protein pairs, there are many instances of interactions between proteins of different functions. But, it is reasonable to assume that interactions that are part of a complex are between similar function protein pairs. In other words, it can be said that the quality of a predicted complex is good if it contains mostly similar function proteins. To examine the quality of the predicted complexes, we estimate the relative amount of interactions that are between similar function protein pairs out of intra-complex interactions for the 20 sets of yeast complexes using (4).

RA=∪i=0nSFIi∪i=0nAIi     (4)
 MathType@MTEF@5@5@+=feaafiart1ev1aaatCvAUfKttLearuWrP9MDH5MBPbIqV92AaeXatLxBI9gBaebbnrfifHhDYfgasaacH8akY=wiFfYdH8Gipec8Eeeu0xXdbba9frFj0=OqFfea0dXdd9vqai=hGuQ8kuc9pgc9s8qqaq=dirpe0xb9q8qiLsFr0=vr0=vr0dc8meaabaqaciaacaGaaeqabaqabeGadaaakeaacqWGsbGucqWGbbqqcqGH9aqpdaWcaaqaamaatadabaGaem4uamLaemOrayKaemysaK0aaSbaaSqaaiabdMgaPbqabaaabaGaemyAaKMaeyypa0JaeGimaadabaGaemOBa4ganiablQIivbaakeaadaWeWaqaaiabdgeabjabdMeajnaaBaaaleaacqWGPbqAaeqaaaqaaiabdMgaPjabg2da9iabicdaWaqaaiabd6gaUbqdcqWIQisvaaaakiaaxMaacaWLjaWaaeWaaeaacqaI0aanaiaawIcacaGLPaaaaaa@48DC@

**Figure 6:**
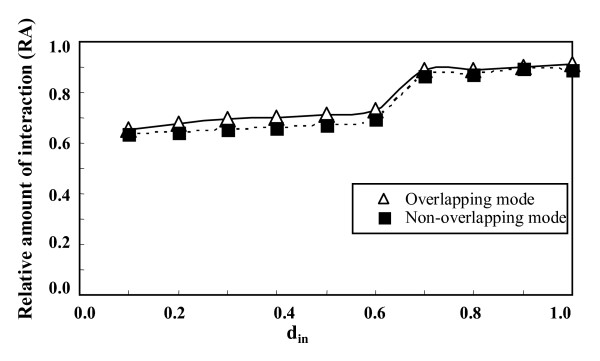
Relative amount of interactions involving similar function protein pairs in 20 sets of yeast complexes against corresponding *d*_*in *_values.

**Figure 7:**
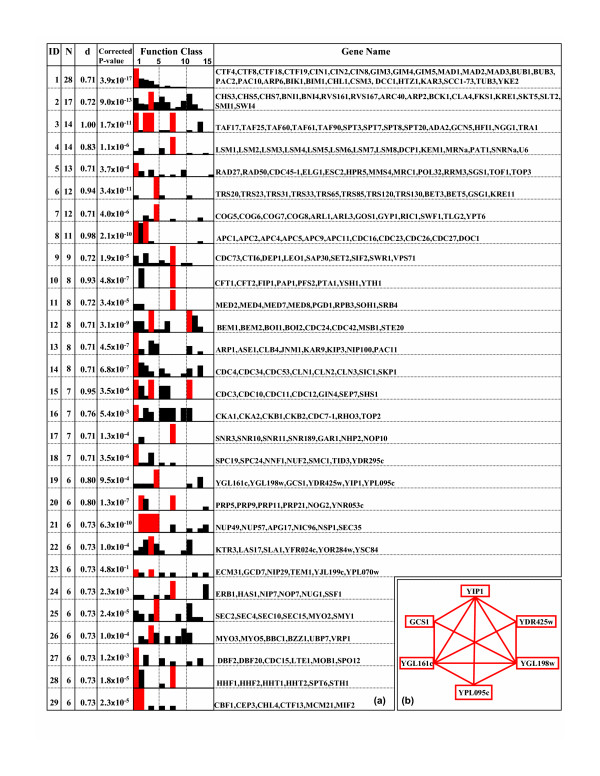
Complexes that are of size ≥ 6 of the set generated using *d*_*in *_= 0.7, *cp*_*in *_= 0.50 and non-overlapping mode. (a) ID, size *N*, density *d*, p-value (with Bonferroni correction), a corresponding histogram and the names of the constituent proteins of each complex. The histogram of a complex shows the distribution of its member proteins with respect to 15 functional classes: (1) Cell cycle and DNA processing, (2) Protein with binding function or cofactor requirement (structural or catalytic), (3) Protein fate (folding, modification, destination), (4) Biogenesis of cellular components, (5) Cellular transport, transport facilitation and transport routes, (6) Metabolism, (7) Interaction with the cellular environment, (8) Transcription, (9) Energy, (10) Cell rescue, defense and virulence, (11) Cell type differentiation, (12) Cellular communication/signal transduction mechanism, (13) Protein activity regulation, (14) Protein synthesis, and (15) Transposable elements, viral and plasmid proteins. A protein may belong to more than one functional class. The scaling of a histogram is according to the size of the corresponding complex. The highest column/columns of a histogram are colored as red. (b) The network of the complex 19 of Fig 7(a).

Here, *n *is the number of complexes of size ≥ 3 in a set, *SFI*_i_ is the number of interactions of cluster *i*, that are between protein pairs of identical functional class, and *Al*_*i *_is the number of all interactions in cluster *i *of the corresponding set. We considered 15 functional classes from MIPS (see the names of these 15 classes in the legend of Fig. 7(a)). Fig. 6 shows the relation between *RA *and *d*_*in*_. There is a sudden rise from *d*_*in *_= 0.6 to *d*_*in *_= 0.7. Thus the complexes having density 0.7 or more have high statistical significance. When complexes are generated using *d*_*in *_= 0.6, many of the complexes consist of three proteins and two interactions (density 0.66). On the other hand when *d*_*in *_= 0.7 is used, the aforementioned type of 3-protein complexes are excluded. Therefore, it may be concluded that many of the interactions contained in 3-protein complexes of density 0.66 are not interactions between similar function protein pairs. The functions of all the proteins involved in the network or complexes are not yet known. It is noticeable that the percentage of interactions between similar function protein pairs is higher in high-density complexes and this percentage might increase if the functions of all the proteins were known. Hence it can be concluded that the interactions that form high-density clusters in PPI networks represent functional complexes and can be considered as true interactions with high probability.

### Details of a group of predicted complexes

In this section, we illustrate the presence of similar function proteins in complexes using specific examples and thus point up that the proposed algorithm can be used for prediction of protein functions. Fig 7(a) presents information on the complexes that are of size ≥ 6 of the set generated using *d*_*in *_= 0.7, *cp*_*in *_= 0.50 and non-overlapping mode. The heights of the columns of a histogram are proportional to the number of proteins of a complex belonging to the corresponding functional classes. A protein may belong to more than one functional class. The highest column/columns of a histogram are colored as red. It is noticeable that in most of the cases the red columns are very near to the maximum possible height indicating that most of the proteins of any complex have one or more common function/functions. To further prove that the accumulation of proteins of a given functional group in a complex did not happen merely by chance, we calculated p-values of these complexes using (5), which is based on hyper geometric distribution.

P=1−∑i=0k−1(Fi)(N−FC−i)(NC)     (5)
 MathType@MTEF@5@5@+=feaafiart1ev1aaatCvAUfKttLearuWrP9MDH5MBPbIqV92AaeXatLxBI9gBaebbnrfifHhDYfgasaacH8akY=wiFfYdH8Gipec8Eeeu0xXdbba9frFj0=OqFfea0dXdd9vqai=hGuQ8kuc9pgc9s8qqaq=dirpe0xb9q8qiLsFr0=vr0=vr0dc8meaabaqaciaacaGaaeqabaqabeGadaaakeaacqWGqbaucqGH9aqpcqaIXaqmcqGHsisldaaeWbqaamaalaaabaWaaeWaaeaafaqabeGabaaabaGaemOrayeabaGaemyAaKgaaaGaayjkaiaawMcaamaabmaabaqbaeqabiqaaaqaaiabd6eaojabgkHiTiabdAeagbqaaiabdoeadjabgkHiTiabdMgaPbaaaiaawIcacaGLPaaaaeaadaqadaqaauaabeqaceaaaeaacqWGobGtaeaacqWGdbWqaaaacaGLOaGaayzkaaaaaiaaxMaacaWLjaWaaeWaaeaacqaI1aqnaiaawIcacaGLPaaaaSqaaiabdMgaPjabg2da9iabicdaWaqaaiabdUgaRjabgkHiTiabigdaXaqdcqGHris5aaaa@4D3E@

Here N, C and F are the sizes of the whole network, a complex and a functional group in the network respectively and k is the number of proteins of the functional group in the complex. The smallest p-value with Bonferroni correction [19] corresponding to each complex is shown in Fig. 7(a) and the very low p-values indicate the statistical significance of the complexes.

Given the fact that proteins of a particular complex are of similar function, if we apply the present algorithm to interaction networks consisting of known and unknown function proteins then it is likely that function-unknown proteins will form cluster with similar and function-known proteins. This may enable us to predict the function of proteins. For example, let us consider the complex 19 of Fig. 7(a), whose network is shown in Fig. 7(b). Protein YDR425w of this complex is related to cellular transport and YIP1, YGL198w, YGL161c and GCS1 are related to vesicular transport. Hence, we predict the function-unknown protein YPL095c of this complex is a transport related protein most likely related to vesicular transport. By analyzing all the generated complexes in a similar way the functions of many other function-unknown proteins can be predicted.

## Conclusion

In this paper we have described an algorithm to detect protein complexes in large interaction networks where a node represents a protein and an edge represents an interaction. We represent the interaction network as an undirected simple graph and then generate clusters in it by ensuring density and checking periphery of the clusters. The input to the algorithm is the associated matrix of an interaction network and the outputs are protein complexes whose densities are more or equal to a threshold value. The worst case timing complexity of the algorithm is polynomial of the order O(|*N*|^3^), where |*N*| is the number of nodes of the network. We show the performance of the algorithm by applying it to two typical PPI networks of *E. coli *and *S. cerevisiae*. We find that similar function proteins usually cluster together which represent molecular biological functional units. Therefore, it is possible to predict the functions of proteins by applying the algorithm to a network that contains both function known and function unknown proteins. A comparison of PPI and random network is also performed in the context of the proposed algorithm and it is observed that the organization of a PPI network is different from that of a random network.

## Methods

The protein interaction data of *E. coli *was collected from [11] and that of the yeast was collected from [12]. Interaction data is generally represented as pairs of interacting proteins or in other words they are the edges of the interaction network. First we discarded self-interactions and generated the adjacency matrix of the entire network. The previously discussed cluster-generating algorithm has been implemented in java programming. It is roughly a 600 line program and this program takes the adjacency matrix of the network and protein list as inputs and generates the clusters. It takes around 20 minutes on an IBM PC with 1.7 GHz processor and 1.5 GB RAM to generate clusters from the PPI network of the yeast, which consists of 4648 proteins and 12487 interactions. The data of the known protein complexes was downloaded from [17]. All the experiments discussed under the section 'Results and Discussion' were performed by java programming. However the high precision p-values were calculated using Python programming.

## Authors' contributions

Md. Altaf-Ul-Amin and Shigehiko Kanaya developed and implemented the clustering algorithm. Yoko Shinbo, Kenji Mihara and Ken Kurokawa designed and performed the experiments. Shigehiko Kanaya supervised the work as a whole.
